# Diffuse Intramuscular Lipomatosis of a Lower Limb

**DOI:** 10.1080/13577149878172

**Published:** 1998-03

**Authors:** Alfred D. Morris, Mike J. Jane, David Ritchie, Tim Helliwell

**Affiliations:** 1 Royal Liverpool and Broadgreen University Hospitals Liverpool UK; 2 Department of Orthopaedic and Trauma Surgery Royal Liverpool University Hospital Prescot Street Liverpool L7 8XP UK

## Abstract

*Patient.* A 40-year-old man presented with a swelling of the left thigh which had been increasing in size over 10 months. Surgery confirmed a diagnosis of lipoma. After 6 months, another swelling appeared, this time in the left calf. Ultrasound-guided biopsies revealed that the tissue showed appearances consistent with intramuscular lipoma. No further surgery was performed and the man is to be reviewed regularly, with possible debulking if necessary.

*Discussion.* This case presents an atypical case of lipomatosis. Magnetic resonance imaging is useful for assessing the extent of the lesion.


*Sarcoma* (1998) **2**, 53–56

CASE REPORT

### Diffuse intramuscular lipomatosis of a lower limb

ALFRED D. MORRIS, MIKE J. JANE, DAVID RITCHIE & TIM HELLIWELL

*Royal Liverpool and Broadgreen University Hospitals, Liverpool, UK*

**Abstract**
*Patient.* A 40-year-old man presented with a swelling of the left thigh which had been increasing in size over 10 months. Surgery confirmed a diagnosis of lipoma. After 6 months, another swelling appeared, this time in the left calf. Ultrasound-guided biopsies revealed that the tissue showed appearances consistent with intramuscular lipoma. No further surgery was performed and the man is to be reviewed regularly, with possible debulking if necessary.
*Discussion.* This case presents an atypical case of lipomatosis. Magnetic resonance imaging is useful for assessing the extent of the lesion.

**Key words**: *intramuscular lipomatosis, magnetic resonance imaging.*

### Introduction

Intramuscular lipoma of the extremities is well recognized, but received little attention until Regan *et al.* in 1946 reported two cases.[1] Intramuscular lipomas are benign tumours, but because of their frequent large size, deep location and infiltrative, unencapsulated growth pattern they can simulate a soft tissue sarcoma. The recommended treatment is complete excision with tumour-free soft tissue margins, with a reported recurrence rate varying from 3% to 62.5%.[2–4] Intramuscular lipoma of the limbs usually occurs in isolated muscles, although one case has been reported where two muscles were involved.[5] Diffuse lipomatosis is a rare condition in which usually large portions of an extremity or the trunk show diffuse overgrowth of mature adipose tissue in the subcutaneous tissue, muscle, fascia and sometimes in bone. This condition is known to affect a single lower limb,[6–8] however, we present a case of progressive diffuse intramuscular lipomatosis afflicting only the musculature of a single lower limb.

### Case history

A 40-year-old gentleman presented with an asymptomatic diffuse swelling over the antero-lateral aspect of his left thigh which had been increasing in size over a period of 10 months. Because of its rapid growth, there was some concern that it may be a soft tissue sarcoma. Magnetic resonance imaging (MRI) of the pelvis and upper thighs showed diffuse intramuscular fatty infiltration present within the entire left tensor fascia lata muscle as well as in the lower portion of gluteus maximus, resulting in overall increase in bulk of the affected musculature (Fig. 1). Less extensive fatty infiltration was also noted within the upper left hamstrings, vastus lateralis and intermedius (Fig. 2). The subcutaneous tissues, remaining pelvic and thigh musculature and the right limb appeared spared. Tissue from an incisional biopsy of the tensor fascia lata showed mature adipose tissue without atypia, consistent with an intramuscular lipoma. As the tissue was from a large mass, the possibility of a well-differentiated liposarcoma could not be completely excluded. This man therefore underwent further surgery to attempt an excisional biopsy. At surgery, there was diffuse lipomatous tissue infiltrating the whole of the extensor compartment, which was only clearable by extensor compartmentectomy. Therefore the lesion was debulked sacrificing the anterior third of tensor fascia lata and part of rectus femoris. Microscopy of the specimen showed mature lipocytes infiltrating muscle in a diffuse manner (Fig. 3). The entrapped skeletal muscle fibres showed atrophy in some areas. There were no lipoblasts or cells with atypical nuclei. The diagnosis of an intramuscular lipoma was confirmed. This man was regularly reviewed in clinic and there was no further swelling of his left thigh. Over the following 6 months, he noticed an asymptomatic generalized swelling of his left calf. MRI showed intramuscular fatty infiltration of the

Correspondence to: M. J. Jane, Department of Orthopaedic and Trauma Surgery, Royal Liverpool University Hospital, Prescot Street, Liverpool, L7 8XP. Tel: +44 151 7064118; Fax: +44 151 7065839.

1357-714X/98/040053-04 ©1998 Carfax Publishing Ltd

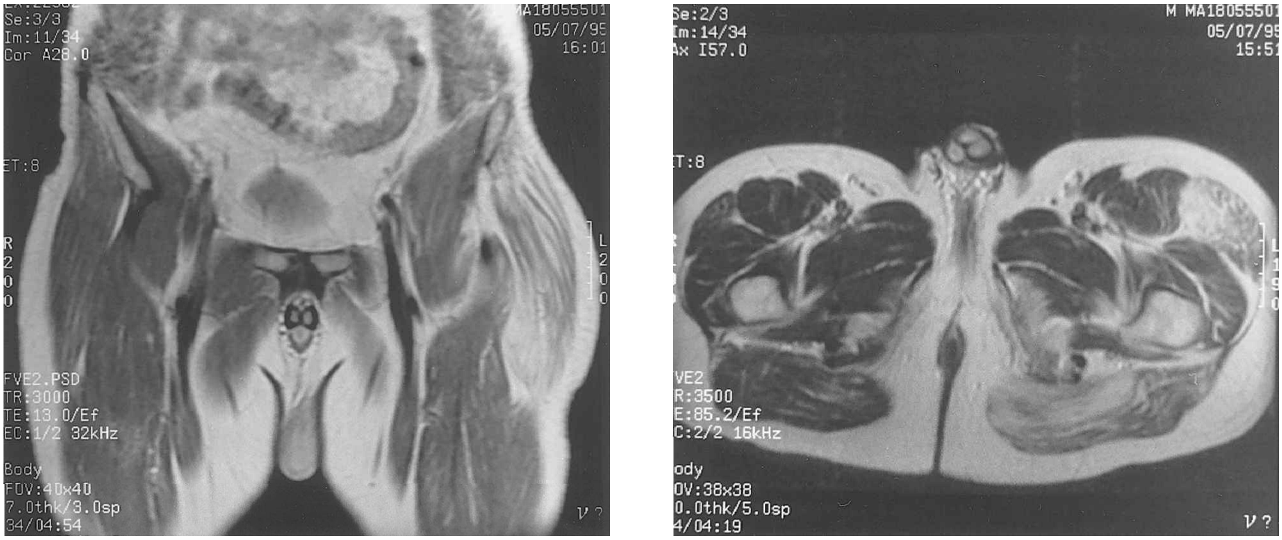

(a) (b)

**Fig. 1.** *Coronal proton density (a) and axial T2-weighted (b) MR images of the upper thighs showing enlargement of the left tensor fascia lata and gluteal muscles due to fatty infiltration.*

lower leg mainly affecting the left soleus and dorsiflexors (Fig. 4). Previous resection of tensor fascia lata was noted, and there was no change in the fatty infiltration of the thigh musculature. The right leg was spared. Ultrasound-guided biopsies of the left soleus were performed and the tissue again showed appearances consistent with an intramuscular lipoma. No further surgery was performed and this gentleman is to be reviewed regularly in the outpatient clinic.

### Discussion

The differential diagnosis of multiple intramuscular lesions includes fibromatosis, metastases, neurofibromatosis, lipomatosis and multiple lipomas. However, a diagnosis of infiltrating lipoma can be suggested by MRI due to its typical signal characteristics and morphology (Fig. 2(b)). Fatty infiltration of muscle also occurs in many neuromuscular disorders but, unlike our case, is usually associated with muscle wasting and loss of muscle volume.[9]

Because of the rapid growth and size of the tumour in this case, soft tissue sarcoma had to be excluded by thorough microscopic examination of the biopsy specimens. Once malignancy had been excluded, the exact nature of surgery must be planned. The main symptoms, if untreated, would be due to increasing size leading to pain, as a result of local pressure on muscle, fascia and nerves, and the effects of compression on neurovascular bun

(a)

(b)

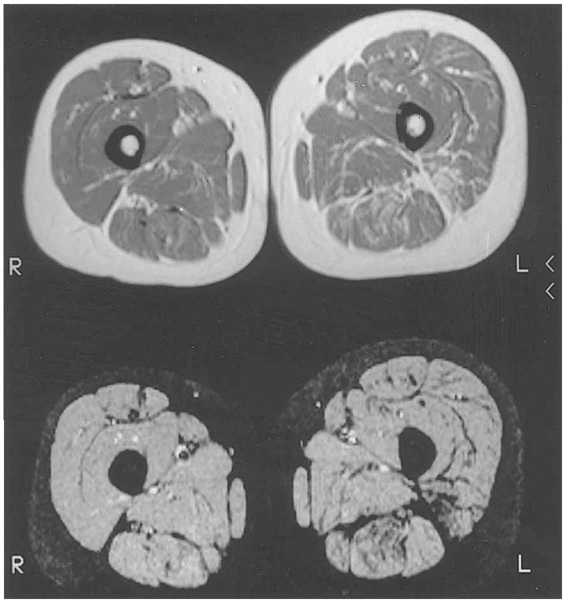

**Fig. 2.** *Axial (a) T1-weighted and (b)short tau inversion recovery (STIR) MR images of the mid thigh confirming further fatty infiltration of the hamstrings, and to a lesser extent vastus lateralis and intermedius. On the STIR sequence the signal intensity of fatty tissue is suppressed giving a low signal thus confirming its diagnosis.*

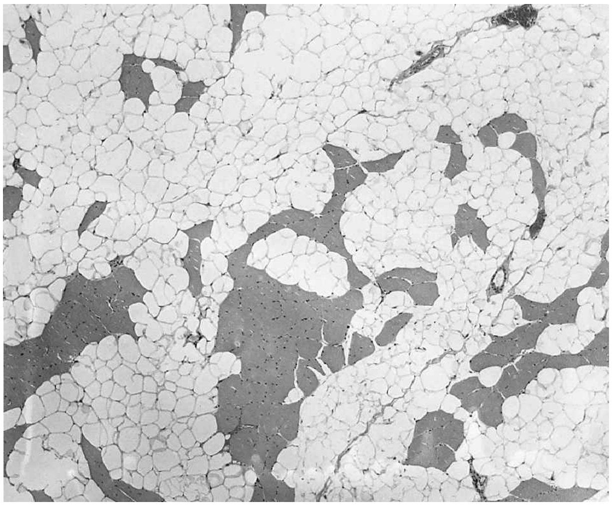

**Fig. 3.** *Histology. This figure is representative of tissue from each of the biopsy specimens, and shows skeletal muscle fibres separated by matu:re adipocytes. Haematoxylin and eosin,* ×*60.*

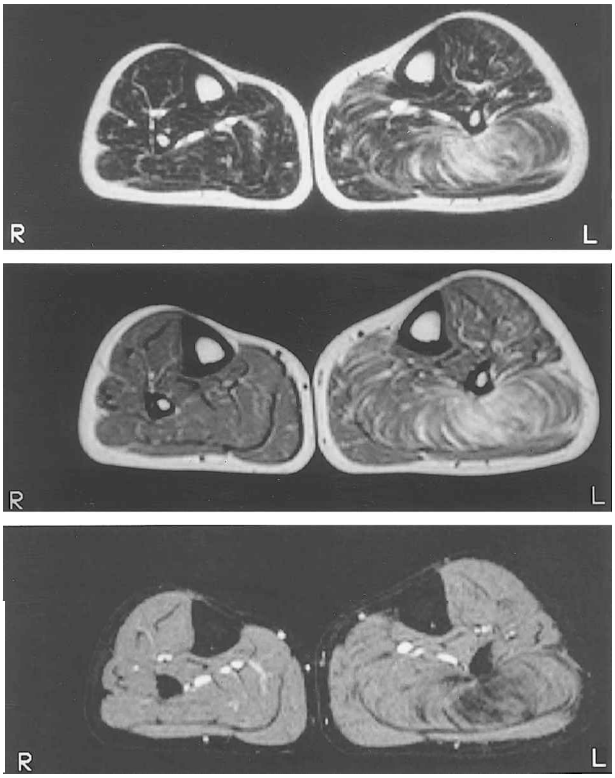

**Fig. 4.** *Axial (a) T1-weighted (b) T2-weighted and (c) short tau inversion recovery (STIR) MR images of mid calf reveals infiltration of the soleus and to a lesser extent the dorsiflexors.*

dles. The only cure is complete excision. However, this would be totally unacceptable in this gentleman because of the resultant disability, for a condition which is benign, where limitation of growth of the lipoma may still occur following partial tumour removal and where the recurrence is usually slow after incomplete excision.[10,11] Once sarcoma was excluded and the tumour debulked, this gentleman required no further surgery as he was asymptomatic. He will require regular follow-up and possible recurrent debulking if he develops symptoms from increasing growth. There is no role for chemotherapy in this condition, and radiotherapy is not advisable as there is a theoretical risk of malignant change.

The histological diagnosis of intramuscular lipoma is straightforward, with separation of skeletal muscle fibres by mature adipose tissue. In large lesions, well-differentiated liposarcoma or atypical intramuscular lipoma are excluded by a lack of cellular atypia or multivacuolated lipoblasts. Primary muscular diseases that may result in fatty replacement of the muscle are excluded by the clinical presentation and the lack of degenerative changes in the muscle fibres. Neurological disorders such as poliomyelitis should also be excluded clinically.

There are various forms of infiltrating lipoma including diffuse lipomatosis, and multiple symmetrical lipomatosis. There are several differences between our case and the more typical form of diffuse lipomatosis:

(1) Diffuse lipomatosis usually presents during the early years of life, although there have been scattered reports of presentation in adulthood.[4]
(2) Diffuse lipomatosis is not limited to the extremities, the trunk and chest wall being commonly involved, although involvement of a single limb has been recorded.[6,8]
(3) Diffuse lipomatosis usually affects the subcutaneous, fascial and osseous structures, including osseous hypertrophy,[4] as well as the musculature, but in this case only the musculature is involved.

Multiple symmetrical lipomatosis typically occurs in middle-aged men but is symmetrical, often involves the trunk and is associated with metabolic disease including liver disease, impaired glucose tolerance, hypertriglyceridaemia and hyperuricaemia.[7] Prolonged stimulation of adipose tissue by adrenal corticosteroids may also lead to overgrowth in several regions of the body.[4] There was no evidence of metabolic disease in this case.

### Summary

(1) This is the first reported case of diffuse intramuscular lipomatosis of one limb, to the best of our knowledge.
(2) Magnetic resonance imaging is useful in the diagnosis and for assessing the extent of this lesion.
(3) Soft tissue sarcoma must be excluded by microscopic examination of biopsy specimens.
(4) Complete excision is unacceptable in this case due to the extent of the lesion, so regular follow-up and possible recurrent debulking may be required if symptoms of compression develop.

### References

1 Regan JM, Bickle WH, Broders AC. Infiltrating benign lipomas of the extremities. *West J Surg* 1946; 54:87–93.
2 Bjerregaard P, Hagen K, Daugaard S, *et al.* Intramuscular lipoma of the lower limb. *J Bone Joint Surg* 1989; 71B(5):812–5.
3 Dionne GP, Seemayer TA. Infiltrating lipomas and angiolipomas revisited. *Cancer* 1974; 33:732–8.
4 Enzinger FM, Weiss SW. *Soft tissue tumours*. St Louis: Mosby, 1995.
5 Greenberg SD, Isensee C, Gonzalez-Angulo A, *et al.* Infiltrating lipomas of the thigh. *Am J Clin Pathol* 1963; 39:66–72.
6 Kindbolm L, Moller-Nielsen J. Diffuse lipomatosis in the leg after poliomyelitis. *Acta Pathol Microbiol Scand* 1975; 83:339–44.
7 Kransdorf MJ, Murphey MD. *Imaging of soft tissue tumors*. Philadelphia: Saunders, 1997.
8 Lippitt DH, Johnston JR. Diffuse lipomatosis of a lower extremity. Report of a case. *Bull Ayer Clinic Penn Hosp* 1954; 4:55–60.
9 Resnick D. *Diagnosis of bone and joint disorders*. Philadelphia: Saunders, 1995.
10 Austin RM, Mack GR, Townsend CM, *et al.* Infiltrating (intramuscular) lipomas and angiolipomas. *Arch Surg* 1980; 115:281–4.
11 Wurlitzer F, Bedrossian C, Ayala A, *et al.* Problems of diagnosing and treating infiltrating lipomas. *Am Surg* 1973; 39:240–3.
